# Biomechanical evaluation of four surgical techniques for ventral stabilization of the atlantoaxial joint in dogs

**DOI:** 10.1590/acb383223

**Published:** 2023-09-18

**Authors:** Danyelle Rayssa Cintra Ferreira, Luís Gustavo Gosuen Gonçalves Dias, Bruno Watanabe Minto, Thiago André Salvitti de Sá Rocha, Caio Afonso dos Santos Malta, Maria Eduarda Bastos Andrade Moutinho da Conceição, Dayvid Vianêis Farias de Lucena

**Affiliations:** 1 Universidade Estadual Paulista “Júlio de Mesquita Filho” – Faculdade de Ciências Agrárias e Veterinárias – Department of Veterinary Medicine and Surgery – Jaboticabal (São Paulo) – Brazil.; 2 Universidade Federal de Jataí – Department of Veterinary Medicine – Jataí (Goiás) – Brazil.

**Keywords:** Atlanto-Axial Joint, Axis, Cervical Vertebra, Cervical Atlas, Dogs, Odontoid Process, Printing, Three-Dimensional

## Abstract

**Purpose:**

This study compared, through biomechanical evaluation under ventral flexion load, four surgical techniques for ventral stabilization of the atlantoaxial joint in dogs.

**Methods:**

In total, 28 identical atlantoaxial joint models were created by digital printing from computed tomography images of a dog, and the specimens were divided into four groups of seven. In each group, a different technique for ventral stabilization of the atlantoaxial joint was performed: transarticular lag screws, polyaxial screws, multiple screws and bone cement (polymethylmethacrylate–PMMA), and atlantoaxial plate. After the stabilization technique, biomechanical evaluation was performed under ventral flexion load, both with a predefined constant load and with a gradually increasing load until stabilization failure.

**Results:**

All specimens, regardless of stabilization technique, were able to support the predefined load without failing. However, the PMMA method provided significant more rigidity (p ≤ 0.05) and also best resisted the gradual increase in load, supporting a significantly higher maximum force (p ≤ 0.05). There was no statistical difference in flexural strength between the transarticular lag screws and plate groups. The polyaxial screws method was significantly less resistant to loading (p ≤ 0.05) than the other groups.

**Conclusions:**

The PMMA technique had biomechanical advantages in ventral atlantoaxial stabilization over the other evaluated methods.

## Introduction

Atlantoaxial subluxation (AAS) occurs as a result of instability, characterized by the dorsal displacement of the cranial portion of the axis body towards the vertebral canal. It can cause variable compression of the spinal cord and its nerve roots^1^-^3^. AAS is usually a consequence of congenital and/or developmental abnormalities in young toy or miniature breed dogs^3^-^8^. However, traumatic fracture of the odontoid process and/or rupture of ligaments can occur in dogs of any breed and age^2^,^7^,^9^-^11^.

The ventral flexion load is an important vector of the atlantoaxial joint in dogs, and the local ligaments that support the weight of the head in the sagittal plane are under continuous tension^2^. Rupture of the ligaments of the atlantoaxial joint has been reported to result in increased mobility, leading to AAS and spinal cord compression^2^.

Surgical treatment is recommended for most dogs with AAS, and many stabilization methods have been reported, including dorsal and ventral access techniques^11^-^16^. Ventral approaches are widely used, since they allow the debridement of the articular cartilage and the placement of bone grafts to promote arthrodesis, as well as inspection and removal of the odontoid process in cases of fracture, non-union or excessive dorsal displacement^4^,^7^,^9^,^17^-^19^. The ventral procedures described in the literature include transarticular implants, pins or screws distributed along the vertebral bodies of C1 and C2 with or without polymethylmethacrylate (PMMA), plate placement or a combination of techniques^1^,^5^,^10^,^20^-^25^.

Although there are many descriptions of AAS stabilization techniques, few biomechanical studies have been performed^3^,^8^,^26^, so there is little information to guide the neurosurgeon in choosing the safest atlantoaxial stabilization technique. The aim of this study was to compare, through biomechanical evaluation under ventral flexion load, four surgical techniques for ventral stabilization of the atlantoaxial joint in dogs: transarticular lag screws, polyaxial screws, multiple screws and PMMA, and a new atlantoaxial plate.

## Methods

This study was carried out with the consent of the Ethics Committee on the Use of Animals at our institution, under the number 007869/18.

### Specimen creation

The template for the specimens was created from computed tomography (CT) images of the first two cervical vertebrae of a 2-year-old mixed breed dog with a body weight of 29 kg. After obtaining the CT images, the vertebral models were sent to computer programs, in which they were virtually reconstructed, and the odontoid process was removed from the axis, generating a specimen with AAS due to odontoid agenesis or fracture.

In the virtual environment, two fixation blocks were created, cranial to C1 and caudal to C2, to fix the specimen in the biomechanical testing machine. In addition, removable dorsal connections between the atlas and the axis were designed, which were intended to maintain the alignment and provide temporary stabilization of the subluxation during insertion of the implants. Thus, the reduction techniques could be standardized for all experimental groups, avoiding implantation errors due to poor vertebral alignment. After the implants had been placed, these connections were removed, and the vertebrae were kept stabilized only by the fixation method for each group.

**Figure 1:**
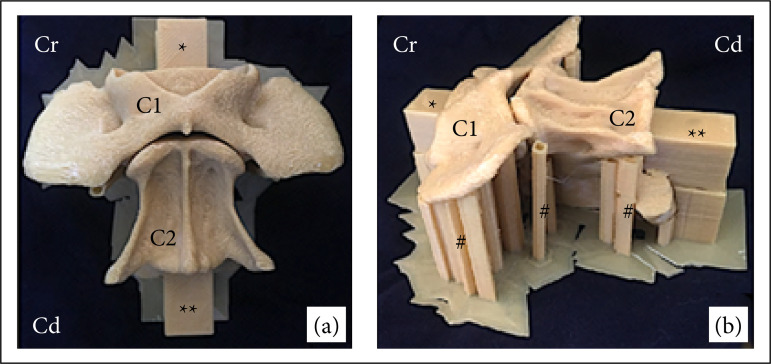
Photographic images of the specimen created by digital printing from computed tomography images representing the ventral aspect of the first and second cervical vertebrae of a dog. **(a)** Ventral view; **(b)** oblique view.

Source: Elaborated by the authors. C1: first cervical vertebra (atlas); C2: second cervical vertebra (axis); *cranial fixation block; **caudal fixation block; #removable dorsal connections between the atlas and the axis; Cr: cranial; Cd: caudal.

The three-dimensional (3D) model of the specimen was rendered, and the specimens printed in 0.3-mm layers with 100% fill of polylactic acid (PLA) by a 3D printer (custom PRUSA i3 model) (Fig. 1). The 28 specimens were divided into the four proposed experimental groups, resulting in seven identical specimens for each surgical technique.

### Atlantoaxial three-dimensional drill guides

Specific atlantoaxial 3D drill guides (3DDG) were made for three of these four techniques (transarticular lag screws, polyaxial screws and multiple screws, and PMMA), to improve precision and standardization of drilling.

For the creation of each guide, the virtual vertebral models obtained from the CT images were used to plan the ideal trajectory of each drill hole, aiming to cover the largest bone stock available and provide the ideal angle. Thus, the virtual drill guide ensured accurate implant placement for each stabilization technique. The contact surface of this guide was constructed to align with the anatomy of the ventral surface of the vertebra to be drilled, ensuring the ideal trajectory. The data from the virtual guide were printed by the direct light processing/stereolithography method, using 405 nanometer resin.

**Figure 2:**
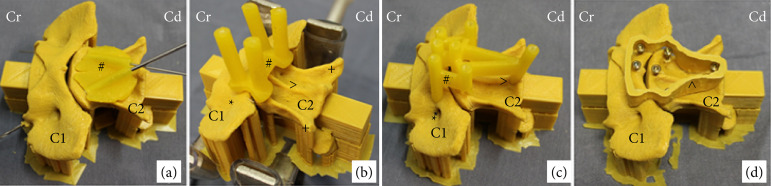
Photographic images of the atlantoaxial three-dimensional drill guides (3DDG) and the mold to accommodate the polymethylmethacrylate (PMMA). **(a)** 3DDG for the transarticular screws group (ventrolateral view); **(b)** 3DDG for the polyaxial screws group (ventrocaudal view); **(c)** 3DDG for the multiple screws and PMMA group (ventrolateral view); **(d)** mold to accommodate the PMMA (ventrolateral view).

Source: Elaborated by the authors. C1: first cervical vertebra (atlas); C2: second cervical vertebra (axis); *C1 transverse foramen; +C2 transverse process; >ventral crest of C2; #atlantoaxial three-dimensional drill guides; ^mold to accommodate the PMMA; Cr: cranial; Cd: caudal.

The 3DDG for the transarticular lag screws group (TA3D) had two drilling holes, each one directed to an atlantoaxial articular surface (Fig. 2a). The 3DDG for the polyaxial screws group (PAX3D) had two drilling holes in the atlas, each one located in the medial aspect of the wing, and two drilling holes in the axis, each one located at the base of the transverse process (Fig. 2b). Finally, the 3DDG for the multiple screws and PMMA group (M3D) had three drilling holes along the caudal aspect of the ventral surface of C1, three drilling holes along the cranial aspect of the ventral surface of C2, and one hole in the caudal aspect of the ventral crest of C2 (Fig. 2C).

Additionally, a mold was printed to accommodate the PMMA used in the stabilization technique with multiple screws and PMMA. The intention was that it would be positioned on the ventral surfaces of C1 and C2, accommodate all the screws and serve as a bed to limit bone cement spillage before polymerization, to ensure that standardized PMMA models would be created in all specimens in this group (Fig. 2d).

### Implants

To perform the stabilization technique with transarticular lag screws, two 2.7-mm diameter titanium cortical screws (Focus Ortopedia, Indaiatuba, São Paulo, Brazil) were used. The polyaxial screw technique required the use of four 2.7-mm diameter titanium polyaxial screws (Focus Ortopedia, Indaiatuba, São Paulo, Brazil), two titanium connecting rods (4 mm in diameter and 40 mm in length) (Focus Ortopedia, Indaiatuba, São Paulo, Brazil) and four counter-screws (Focus Ortopedia, Indaiatuba, São Paulo, Brazil). In the technique with multiple screws and PMMA, seven 2.7-mm diameter titanium cortical screws (Focus Ortopedia, Indaiatuba, São Paulo, Brazil) and a standardized amount of PMMA (12-g powdered polymer and 6-mL of liquid monomer) (VIPI Flash, VIPI Indústria, Comércio, Exportação e Importação de Produtos Odontológicos LTDA, São Paulo, Brazil, lot 0000165034 of powdered polymer and lot 0000168852 of liquid monomer) were used.

For the atlantoaxial plate technique, a titanium locking plate (Focus Ortopedia, Indaiatuba, São Paulo, Brazil) was custom designed so that the screws assumed ideal locations and angulations, to provide a stable and secure fixation. The plate had a butterfly design, being slightly contoured to follow the morphology of the vertebrae. It had four holes with a threaded locking system, which received 2.7-mm diameter titanium locked screws (Focus Ortopedia, Indaiatuba, São Paulo, Brazil). Two holes delivered screws into the medial aspect of the wings of the atlas, and the other two into the base of the transverse process of the axis.

### Surgical techniques for ventral atlantoaxial stabilization

**Figure 3:**
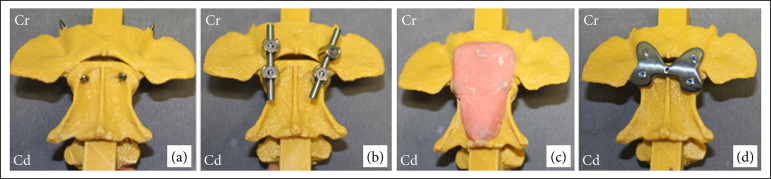
Photographic images of a specimen of each group after the stabilization technique (ventral aspect). **(a)** Transarticular lag screws; **(b)** polyaxial screws; **(c)** multiple screws and polymethylmethacrylate; **(d)** atlantoaxial plate.

Source: Elaborated by the authors. Cr: cranial; Cd: caudal.

In the transarticular lag screws method, the specific drill guide (TA3D) was used. Through it, the axis and the atlas were drilled to allow the implantation of a cortical screw with a compressive effect on each articular surface (Fig. 3a).

In the polyaxial screws method, the PAX3D was used, and, through it, the four bicortical perforations were performed. A polyaxial screw was placed in each hole, and a rod was placed connecting the ipsilateral screws between the atlas and the axis, and, finally, the counter-screws were tightened (Fig. 3b).

In the multiple screws and PMMA method, the M3D was used. In the atlas and in the cranial aspect of the axis, the lateral drill holes were bicortical, whereas the central ones were monocortical. The drill hole in the ventral crest of C2 was similarly monocortical. Thus, the cortical screws could be implanted in their respective holes, so that they protruded about 5 mm from the vertebral surface and were used to fix the bone cement. Finally, the PMMA mold was fitted around the screws and filled with bone cement. This mold was removed a few seconds before complete polymerization of the PMMA (Fig. 3c).

In the atlantoaxial plate method, the new implant was positioned on the vertebral surfaces, and the perforation guides were threaded into the plate holes, directing the bicortical drill holes and allowing the subsequent implantation of the locked screws (Fig. 3d).

After each technique was complete, the temporary dorsal connections of the specimens were removed, leaving the vertebral models stabilized only by the fixation method applied.

### Biomechanical tests

The biomechanical tests were divided into two phases. First, the weight-bearing capacity of each fixation method was evaluated, obtaining values of stiffness (N/mm) and deflection (mm). To this end, the head and atlas of a thawed dog cadaver with similar C1 and C2 dimensions to those of the specimen were weighed. The dog had died of natural causes not related to this study. The distance from the atlantoaxial joint to the center of the head was also measured on this same cadaver, and this value was used as the standard clearance distance in the biomechanical testing machine. The head weight was 2.5 kg (25 N), and the distance was 90 mm.

Thus, the axis fixation block was attached to the universal testing machine (EMIC, model DL 100,000, equipped with a 200 kgf load cell), and a 25-N load was applied in the ventral direction on the atlas fixation block, representing the ventral flexion force. The bending test speed was 5 mm/min, and the free span distance was 90 mm. The machine, together with the Tesc software version 3.04, produced the values of the configured biomechanical properties and a force × deformation graph. All specimens were tested individually.

The second phase of the tests was methodologically similar to the first one, differing only in that the load was applied gradually to the C1 fixation block. The load was increased until there was failure of the stabilization (breakpoint), and the maximum force (N) supported before deformation, and the location of this failure was recorded in each test model.

### Statistical analysis

Statistical analysis was performed using the GraphPad Prism 5 software (GraphPad Software Inc., San Diego, California, United States of America). Initially, the normality of the residuals (Shapiro-Wilk’s test) and homoscedasticity of the variances (Bartlett’s test of variance) of all parameters studied were tested. Means between groups were compared by analysis of variance (ANOVA) and analysis between pairs with Tukey’s post-test. The significance was fixed at p ≤ 0.05 for all tests.

## Results

**Figure 4:**
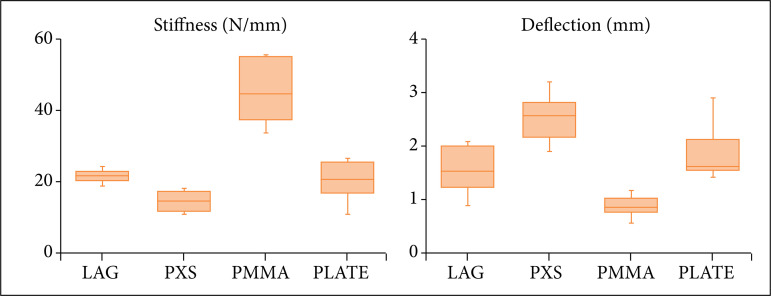
Boxplots representing the quartiles of stiffness (N/mm) and deflection (mm) values for the transarticular lag screws (LAG), polyaxial screws (PXS), multiple screws and PMMA (PMMA), and atlantoaxial plate (PLATE) when subjected to a load of 25 N in the ventral direction.

Source: Elaborated by the authors.

In the first phase of the biomechanical tests, all four methods supported the load without failing. The method of multiple screws and PMMA proved to be significantly more rigid (p ≤ 0.05) than the other stabilization techniques. There was no significant difference (p > 0.05) in stiffness between the other three techniques (Table 1 and Fig. 4). The specimens stabilized with multiple screws, and PMMA suffered significantly less deflection (p≤0.05). That is, the displacement (in mm) between atlas and axis, when subjected to a load of 25 N, was less than for all other techniques. The deflection in the transarticular lag screws group was significantly lower (p ≤ 0.05) than the deflection in the polyaxial screws group and with no statistical difference (p > 0.05) in the deflection in the atlantoaxial plate (Table 2 and Fig. 4).

**Table 1:** Mean and standard deviation of stiffness (N/mm) of specimens when subjected to a load of 25 N in the ventral direction, representing the ventral flexion force*.

Surgical technique	Mean (± SD) of stiffness (N/mm) under load of 25N
Transarticular lag screws	21.72 ± 7.36 ^B^
Polyaxial screws	14.50 ± 2.75 ^B^
Multiple screws and bone cement	45.74 ± 8.29 ^A^
Atlantoaxial plate	20.32 ± 5.56 ^B^
Inter-group comparisons	p-value interval
Multiple screws and bone cement/transarticular lag screws	0.0001 to 0.001 (extremely significant)
Multiple screws and bone cement/polyaxial screws	0.0001 to 0.001 (extremely significant)
Multiple screws and bone cement/atlantoaxial plate	0.0001 to 0.001 (extremely significant)

Source: Elaborated by the authors. SD: standard deviation; *different letters demonstrate differences between the techniques (p≤0.05) by Tukey’s test.

**Table 2:** Mean and standard deviation of deflection (mm) of specimens when subjected to a load of 25 N in the ventral direction, representing the ventral flexion force*.

Surgical technique	Mean (± SD) of deflection (mm) under load of 25N
Transarticular lag screws	1.50 ± 0.42 ^B,a^
Polyaxial screws	2.46 ± 0.43 ^B,b^
Multiple screws and bone cement	0.83 ± 0.19 ^A^
Atlantoaxial plate	1.87 ± 0.52 ^B,a^
Inter-group comparisons	p-value interval
Multiple screws and bone cement/transarticular lag screws	0.01 to 0.05 (significant)
Multiple screws and bone cement/polyaxial screws	0.0001 to 0.001 (extremely significant)
Multiple screws and bone cement/atlantoaxial plate	0.0001 to 0.001 (extremely significant)
Transarticular lag screws/polyaxial screws	0.001 to 0.01 (very significant)

Source: Elaborated by the authors. SD: standard deviation; *Different letters demonstrate differences between the techniques (p≤0.05) by Tukey’s test.

**Figure 5:**
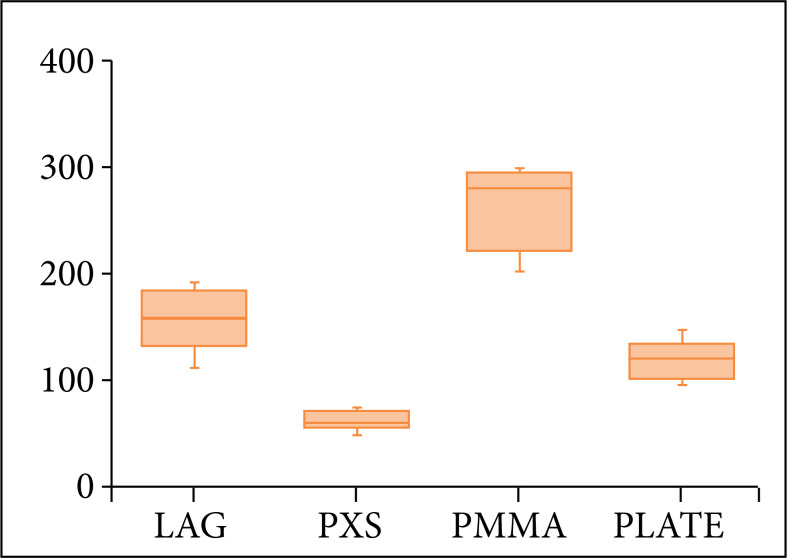
Boxplot representing the quartiles of maximum force **(N)** values for the transarticular lag screws (LAG), polyaxial screws (PXS), multiple screws and PMMA (PMMA), and atlantoaxial plate (PLATE) under ventral flexion force.

Source: Elaborated by the authors.

The method of multiple screws and PMMA supported a significantly higher maximum load (p ≤ 0.05) than all other groups. The maximum force supported by the transarticular lag screws method was significantly higher (p ≤ 0.05) than that of the polyaxial screws, but there was no statistical difference (p > 0.05) in the maximum force supported by the new atlantoaxial plate group and the transarticular lag screws method. The maximum force supported by the new atlantoaxial plate was also significantly higher (p ≤ 0.05) than the maximum load supported by the group of polyaxial screws (Table 3 and Fig. 5).

**Table 3:** Mean and standard deviation of the maximum force **(N)** supported by the specimens under ventral flexion force*.

Surgical technique	Mean (± SD) of the maximum force (N)
Transarticular lag screws	156.4 ± 28.69 ^B,a^
Polyaxial screws	62.27 ± 9.26 ^B,b^
Multiple screws and bone cement	264.18 ± 78.85 ^A^
Atlantoaxial plate	118.87 ± 17.53 ^B,a^
Inter-group comparisons	p-value interval
Multiple screws and bone cement/transarticular lag screws	0.0001 to 0.001(extremely significant)
Multiple screws and bone cement/polyaxial screws	0.0001 to 0.001(extremely significant)
Multiple screws and bone cement/atlantoaxial plate	0.0001 to 0.001(extremely significant)
Transarticular lag screws/polyaxial screws	0.0001 to 0.001(extremely significant)
Atlantoaxial plate/ polyaxial screws	0.001 to 0.01(very significant)

Source: Elaborated by the authors. SD: standard deviation; *different letters demonstrate differences between the techniques (p ≤ 0.05) by Tukey’s test.

Failures in the group of transarticular lag screws were mainly characterized by fracture of the cranial portion of the axis, at the site of implantation of the screws. In the group of multiple screws and PMMA, all failures occurred due to fracture of the bone cement in the joint space between atlas and axis. In the atlantoaxial plate and polyaxial screws methods, there was an increase in the dorsal atlantoaxial distance in the specimens at the end of the biomechanical tests, especially in the latter technique. In the polyaxial screws group, there were a final atlantoaxial distance between 8 and 16 mm and a gap in the locking system between the screw and the rod, while in the atlantoaxial plate group the final atantoaxial distance ranged from 2 to 8 mm.

## Discussion

A biomechanical evaluation of the effect of ventral flexion force was carried out on four methods of ventral stabilization of the atlantoaxial joint in dogs: transarticular lag screws, polyaxial screws, multiple screws and PMMA, and atlantoaxial plate. All methods were able to support a constant load representing the weight of a dog’s head, but the use of multiple screws and PMMA provided significantly more rigidity than all other evaluated methods. When a gradual load was applied until the failure of the construct, the cemented technique supported a significantly higher maximum load than the other methods, while the polyaxial screws group supported a significantly lower maximum load compared to the other groups.

Leblond et al.^26^ also evaluated the rigidity of a few stabilization methods under physiological conditions. They found no significant difference between their three experimental groups (transarticular screws and two different cemented constructions), whereas in the present study the cemented construction proved to be significantly more rigid than the other methods, although all four techniques were able to support the predefined load without failing. The absolute values cannot be directly compared between two studies, because the use of different methodological parameters produced results with different measurement units which do not allow direct comparison.

The second phase of the biomechanical tests evaluated the flexural strength of each fixation method when subjected to high loads, determining the maximum strength supported by the construct before failure. Our results can be more directly compared with those of Takahashi et al.^8^, who also measured the mean maximum load of flexural strength test in each of their atlantoaxial fixation group (atlantoaxial plate fixation, PMMA fixation and transarticular fixation). Despite the use of cadaveric bone specimens by Takashahi et al.^8^, as opposed to the use of PLA specimens in our study, surprisingly the transarticular screw technique achieved similar mean maximum force between the two studies, being 159.5 ± 41.1 N and 156.4 ± 28.69 N, respectively. Therefore, the structural properties of PLA were not limiting for the successful biomechanical evaluation of the atlantoaxial stabilization techniques in our study. Moreover, there was less variability of the samples than with the use of cadaveric specimens, since the standard deviation of our sample group was lower than the standard deviation of the study by Takahashi et al.^8^.

Still in this comparative context, the average maximum force achieved by the plate group in the study by Takahashi et al.^8^ was 280 ± 14.1 N, in contrast to our average maximum force, of 118.87 ± 17.53 N. This disparity in results is thought to be attributable to two main reasons. First, the use of transarticular pins in association with the plate in the technique by Takahashi et al.^8^, provided greater rigidity of the construct. In addition, the plate model used by Takahashi et al.^8^ has a greater contact surface on the vertebral bodies of C1 and C2, unlike our plate, which is concentrated in the ventral arch of the atlas and in the cranial portion of the axis. Finally, when comparing the screw and bone cement techniques between the two studies, there were higher mean maximum force values in the study by Takahashi et al.^8^ compared to ours, being, respectively, 510 ± 122.3 N and 264.18 ± 78 N. This difference can also be attributed to the use of transarticular pins in association with screws and bone cement in the technique by Takahashi et al.^8^, leading to greater stability of the fixation and may also be a bias in the biomechanical evaluation of the isolated technique.

The long-term goal of ventral atlantoaxial stabilization is arthrodesis^27^. The general principles of any arthrodesis are removal of articular cartilage, grafting, apposition of the articular surfaces at the correct angulation, and rigid stabilization^28^. Thus, if these principles are applied to all techniques and only the method of stabilization varies, the more rigid stabilizations should provide better results. This reasoning suggests that, among the techniques tested, multiple screws and PMMA may have the potential to provide the best results in atlantoaxial fusion. Clinical data is nevertheless necessary to support this statement.

In our study, it is likely that the specific placement of the implants is responsible for the biomechanical advantages of the cemented construction, as the force concentration point of the atlantoaxial region tends to be located in the cranial portion of the axis^29^,^30^. The presence of screws and bone cement along the entire surface of the vertebral body of C2 better distributes the loads transmitted in this region, making the cemented construct better able to support flexion loads^26^. The implants in the configurations of transarticular lag screws, atlantoaxial plate and polyaxial screws are concentrated in the cranial portion of the axis, in which forces are concentrated, and thus bear significantly lower loads.

The maximum force supported by the transarticular lag screws group did not differ significantly from the atlantoaxial plate group, but both supported significantly higher maximum force than the polyaxial screws group. The biomechanical properties of each construct may help explain these results.

In the transarticular lag screws technique, the implants were positioned with a compressive effect (compressing the articular surfaces of the axis and the atlas). Thus, there was load sharing between the implant and the specimen, reducing the load supported by screws and increasing fixation stability.

However, the configurations obtained with the atlantoaxial plate and polyaxial screws lacked such a load-sharing mechanism, as these constructs had their implants firmly attached to the ventral arch of the atlas and the cranial portion of the axis, without direct compression between the joint surfaces. Thus, according to the principles of internal fixation^31^, load transfer occurred only through the implants. The atlantoaxial plate group provided satisfactory stabilization in this region, supporting a similar maximum load to the group of transarticular lag screws, which was almost double the maximum load supported by the polyaxial screws group.

Finally, the group of polyaxial screws may have supported a significantly lower maximum load than all the other methods due to the fragility of its locking system. Studies have shown that the design of some polyaxial locking mechanisms makes them more vulnerable to failure^32^,^33^. In the present study, the construction did not support high flexion loads, as there was a gap in the locking system between the screw and the rod, allowing the rod to move inside the device resulting in the failure of the construction. This instability led to excessive atlantoaxial distancing, as the screw was no longer locked in the system. When load was applied, the distancing was not contained by any effective stabilization, and atlantoaxial displacements were at least 8 mm, practically double the distance recognized as diagnostic for AAS.

The methodology of this study has some limitations. First, the specimens used as experimental models were significantly larger than the vertebral size of toy breed dogs typically affected by AAS. However, the techniques used, the implant placement sites and the comparison between groups did not seem to be limiting factors for the applicability of these results in smaller dogs. Furthermore, dogs of any age, size and breed can be affected by traumatic AAS. In addition, previous biomechanical studies involving atlantoaxial stabilization use Beagle dogs as models^3^,^8^,^26^. So, our study allows for more reliable comparisons between the results.

The biomechanical evaluation was performed only under ventral flexion force, but the atlantoaxial joint of a live animal is submitted to multidirectional movements. Although studying effects of a unidirectional force is a possible limitation, this ventral flexion force is considered one of the main vectors in AAS since the weight of the head is the most significant source of tension in the canine atlantoaxial joint^2^,^3^. Finally, this study was carried out under *in-vitro* conditions, and the effects of clinical factors cannot be discussed.

## Conclusion

Based on the experimental results, we concluded that the better distribution of the implants along the vertebral surfaces of C1 and C2 provides biomechanical advantages, such that the cemented technique is superior, both under the action of predefined constant load and a gradual increase in load, and the polyaxial screws technique, with the configuration proposed in this study, has no biomechanical advantages for atlantoaxial stabilization in PLA models.

## Data Availability

All data sets were generated or analyzed in the current study.

## References

[1] Platt SR, Chambers JN, Cross A. (2004). A modified ventral fixation for surgical management of atlantoaxial subluxation in 19 dogs. Vet Surg.

[2] Reber K, Bürki A, Reves NV, Stoffel M, Gendron K, Ferguson SJ, Forterre F. (2013). Biomechanical evaluation of the stabilizing function of the atlantoaxial ligaments under shear loading: a canine cadaveric study. Vet Surg.

[3] Riedinger B, Bürki A, Stahl C, Howard J, Forterre F. (2015). Biomechanical evaluation of the stabilizing function of three atlantoaxial implants under shear loading: a canine cadaveric study. Vet Surg.

[4] Shores A, Tepper LC. (2007). A modified ventral approach to the atlantoaxial junction in the dog. Vet Surg.

[5] Aikawa T, Shibata M, Fujita H. (2013). Modified ventral stabilization using positively threaded profile pins and polymethylmethacrylate for atlantoaxial instability in 49 dogs. Vet Surg.

[6] Sánchez-Masian D, Luján-Feliu-Pascual A, Font C, Mascort J. (2014). Dorsal stabilization of atlantoaxial subluxation using non-absorbable sutures in toy breed dogs. Vet Comp Orthop Traumatol.

[7] Stalin C, Gutierrez-Quintana R, Faller K, Guevar J, Yeamans C, Penderis J. (2015). A review of canine atlantoaxial joint subluxation. Vet Comp Orthop Traumatol.

[8] Takahashi F, Hakozaki T, Kanno N, Harada Y, Yamaguchi S, Hara Y (2017). Biomechanical evaluation of three ventral fixation methods for canine atlantoaxial instability: a cadaveric study. J Vet Med Sci.

[9] Beaver DP, Ellison GW, Lewis DD, Goring RL, Kubilis PS, Barchard C. (2000). Risk factors affecting the outcome of surgery for atlantoaxial subluxation in dogs: 46 cases (1978-1998). J Am Vet Med Assoc.

[10] Sanders SG, Bagley RS, Silver GM, Moore M, Tucker RL (2004). Outcomes and complications associated with ventral screws, pins, and polymethyl methacrylate for atlantoaxial instability in 12 dogs. J Am Anim Hosp Assoc.

[11] Forterre F, Revés NV, Stahl C, Gendron K, Spreng D. (2012). An indirect reduction technique for ventral stabilization of atlantoaxial instability in miniature breed dogs. Vet Comp Orthop Traumatol.

[12] McCarthy RJ, Lewis DD, Hosgood G (1995). Atlantoaxial subluxation in dogs. Compend Contin Educ Pract Vet.

[13] Jeffery ND (1996). Dorsal cross pinning of the atlantoaxial joint: new surgical technique for atlantoaxial subluxation. J Small Anim Pract.

[14] Lorigados CAB, Sterman FA, Pinto AC. (2004). Clinic-radiographic study of congenital atlantoaxial subluxation in dogs. Braz J Vet Res An Sci.

[15] Havig ME, Cornell KK, Hawthorne JC, McDonnell JJ, Selcer BA. (2005). Evaluation of nonsurgical treatment of atlantoaxial subluxation in dogs: 19 cases (1992-2001). J Am Vet Med Assoc.

[16] Pujol E, Bouvy B, Omaña M, Fortuny M, Riera L, Pujol P. (2010). Use of the Kishigami atlantoaxial tension band in eight toy breed dogs with atlantoaxial subluxation. Vet Surg.

[17] Schulz KS, Waldron DR, Fahie M. (1997). Application of ventral pins and polymethylmethacrylate for the management of atlantoaxial instability: results in nine dogs. Vet Surg.

[18] Revés NV, Stahl C, Stoffel M, Bali M, Forterre F. (2013). CT scan-based determination of optimal bone corridor for atlantoaxial ventral screw fixation in miniature breed dogs. Vet Surg.

[19] Slanina MC (2016). Atlantoaxial instability. Vet Clin North Am Small Anim Pract.

[20] Sorjonen DC, Shires PK (1981). Atlantoaxial instability: a ventral surgical technique for decompression, fixation and fusion. Vet Surg.

[21] Denny HR, Gibbs C, Waterman A. (1988). Atlantoaxial subluxation in the dog: a review of thirty cases and evaluation of treatment by lag screw fixation. J Small Anim Pract.

[22] Sorjonen DC, Simpson ST (1991). Surgical management of atlantoaxial subluxation in 23 dogs. Vet Surg.

[23] Stead AC, Anderson AA, Coughlan A (1993). Bone plating to stabilize atlantoaxial subluxation in four dogs. J Small Anim Pract.

[24] Dickimeit M, Alves L, Pekarkova M, Forterre F. (2011). Use of a 1.5 mm butterfly locking plate for stabilization of atlantoaxial pathology in three toy breed dogs. Vet Comp Orthop Traumatol.

[25] Jeserevics J, Srenk P, Beranekl J, Jaggy A, Touru S, Cizinauskas S (2008). Stabilisation of atlantoaxial subluxation in the dog through ventral arthrodesis. Schweiz Arch Tierheilkd.

[26] Leblond G, Moens NMM, Gaitero L, Linden AZ, James FMK, Monteith G, Runciman RJ (2018). Computed tomography and biomechanical comparison between trans-articular screw fixation and 2 polymethylmethacrylate cemented constructs for ventral atlantoaxial stabilization. Vet Comp Orthop Traumatol.

[27] Panjabi MM. (1992). The stabilizing system of the spine. Part II. Neutral zone and instability hypothesis. J Spinal Disord.

[28] Buote NJ, McDonald D, Radasch R (2009). Pancarpal and partial carpal arthrodesis. Compend Contin Educ Vet.

[29] Stone EA, Betts CW, Chambers JN (1979). Cervical fractures in the dog: a literature and case review. J Am Anim Hosp Assoc.

[30] Hawthorne JC, Blevins WE, Wallace LJ, Glickman N, Waters DJ (1999). Cervical vertebral fractures in 56 dogs: a retrospective study. J Am Anim Hosp Assoc.

[31] Gardner MJ, Helfet DL, Lorich DG (2004). Has locked plating completely replaced conventional plating?. Am J Orthop (Belle Mead NJ).

[32] Stanford RE, Loefler AH, Stanford PM, Walsh WR (2004). Multiaxial pedicle screw designs: static and dynamic mechanical testing. Spine (Phila Pa 1976).

[33] Liu T, Zheng WJ, Li CQ, Liu G, Zhou Y. (2010). Design and biomechanical study of a modified pedicle screw. Chin J Traumatol.

